# TGFB2 Expression and Methylation Predict Overall Survival in Pancreatic Ductal Adenocarcinoma Patients

**DOI:** 10.3390/ijms26136357

**Published:** 2025-07-01

**Authors:** Muhammad Wasif Saif, Wen-Han Chang, Scott Myers, Michael Potts, Sanjive Qazi, Vuong Trieu

**Affiliations:** 1 Karmanos Cancer Institute, 4100 John R Street, HW04HO, Detroit, MI 48201, USA; saifw@karmanos.org; 2 Oncotelic Therapeutics, 29397 Agoura Road, Suite 107, Agoura Hills, CA 91301, USA; scott.myers@oncotelic.com (S.M.); michael.potts@sapubio.com (M.P.); sanjive.qazi@sapubio.com (S.Q.); vtrieu3@autotelicinc.com (V.T.)

**Keywords:** PDAC, pancreatic cancer, TGF-β, TGFB2, IL6, methylation, OT-101, trabedersen

## Abstract

Transforming growth factor-beta (TGF-β) exhibits dual roles in pancreatic ductal adenocarcinoma (PDAC), acting as a tumor suppressor in early stages and a tumor promoter in later disease. Among the three isoforms, TGFB2 is particularly associated with poor prognosis and aggressive phenotypes. This study evaluated the prognostic significance of TGFB2 mRNA and methylation levels in PDAC, with an emphasis on age-dependent effects. Bioinformatic analyses revealed that high TGFB2 expression was significantly associated with reduced overall survival (OS) in patients under 65 (TGFB2 high vs. low median OS: 17.9 vs. 66.9 months) but not in older cohorts. IL6 expression, a downstream target of TGF-β, followed a similar survival profile. Moreover, elevated *TGFB2* methylation showed improved survival in younger patients (high methylation vs. low methylation median OS: 66.9 vs. 17.9 months). In addition, our clinical data from a PDAC trial using OT-101, an antisense oligonucleotide targeting TGFB2, further supported these findings—young patients treated with OT-101 showed improved OS compared to untreated controls. Notably, the methylation of *TGFB1* also correlated with better OS in young patients. These results demonstrate the importance of TGFB2 as both a prognostic biomarker and therapeutic target in younger PDAC patients and further suggest that epigenetic modulation plays a key role in TGF-β signaling in pancreatic cancer progression. Our study emphasizes the isoform- and age-specific prognostic significance of TGFB2 in PDAC and supports the potential insights provided through methylation and expression profiling for personalized treatment strategies, particularly for younger patients who may benefit most from TGFB2-targeted therapies.

## 1. Introduction

TGF-β is a major contributor to pancreatic cancer [1]. TGF-β plays a paradoxical role as both a tumor suppressor and a tumor promoter in pancreatic cancer [1]. TGF-β signals primarily through transmembrane receptor kinases, activating downstream Smad proteins, allowing them to translocate to the nucleus and participate in the transcriptional control of TGF-β target genes. When intact, this pathway functions as a growth inhibitor for many cell types by inhibiting cell cycle progression, and loss of TGFβ responsiveness can lead to deregulated cell proliferation and ultimately tumor progression [2]. Smad4/DPC4 gene inactivation is particularly frequent in pancreatic cancer, contributing to the highly invasive and metastatic characteristics [3]. In those tumors retaining normal Smad4 function, TGF-β can exert potent antiproliferative effects by inducing cell cycle inhibitors such as p21(WAF1/CIP1) [4]. Despite its tumor-suppressive function in early stages, TGF-β often adopts a tumor-promoting role in the late phase of tumor progression [3]. TGF-β can stimulate fibrosis, facilitate immune evasion, and promote epithelial–mesenchymal transition (EMT) [5,6]. Chronic, high-level TGF-β exposure can drive aggressive tumor phenotype[s] and neoplastic transformation, in part through the downregulation of p21(WAF1/CIP1) [4]. TGF-β may promote pancreatic cancer development by affecting stromal and hematopoietic cell function, fueling immunosuppression and rendering the tumor microenvironment more permissive to tumor growth [6]. Multiple therapies have been investigated to target TGF-β signaling, including small-molecule inhibitors, neutralizing antibodies, and antisense approaches [5,7]. The inhibition of TGF-β2 synthesis via the antisense oligonucleotide trabedersen (AP 12009, OT-101) is a promising approach given that increased TGF-β2 levels in the serum or tumor tissue of patients with pancreatic cancer correlated with poor prognosis [7]. The inhibition of TGF-βR to target components of the tumor microenvironment warrants consideration as a potential therapy for pancreatic cancer, particularly in patients who have already lost tumor-suppressive TGF-β signals in the epithelium [6].

There are three main TGF-β isoforms—TGF-β1, TGF-β2, and TGF-β3. All three isoforms are overexpressed in pancreatic cancer compared to normal pancreatic tissue, but to varying degrees: pancreatic adenocarcinomas showed 11- (*p* < 0.001), 7- (*p* < 0.05), and 9-fold (*p* < 0.001) increases in the messenger RNA (mRNA) levels encoding TGF-β1, TGF-β2, and TGF-β3, respectively [8]. Immunohistochemical analyses revealed TGF-β1 presence in 47% of tumors, TGF-β2 in 42%, and TGF-β3 in 40% [8]. Among the three isoforms, TGF-β2 was particularly associated with advanced tumor stage (*p* < 0.05) and more prolonged postoperative survival [8]. In chronic, cancer-associated obstructive pancreatitis (COP), TGF-β1 was prominent in macrophages and neutrophils, TGF-β2 was intensely positive in ductal cells, and TGF-β3 was broadly and less intensely distributed [9]. These findings suggest that TGF-β1 may often be derived from inflammatory cells, while TGF-β2 and TGF-β3 may be more involved in paracrine signaling within ductal epithelial compartments. All three isoforms can promote pathological fibrosis and tumor progression in pancreatic cancer, in part by stimulating myofibroblasts. TGF-β1, however, shows particularly strong links to inflammatory cell infiltration [9], whereas TGF-β2 has been specifically correlated with higher tumor stages [8]. These distinctions highlight different cellular sources and slightly different roles in driving the fibroinflammatory and invasive phenotypes of pancreatic cancer. Because all three isoforms are frequently overexpressed, attempts at targeting TGF-β pathways (e.g., TGF-β ligands, receptors, or intracellular mediators) might need to account for overlapping yet somewhat distinct functional roles of TGF-β1, TGF-β2, and TGF-β3. Prognostically, the detection of TGF-β2 in particular may be relevant in advanced disease [8], though further mechanistic studies are required to delineate each isoform’s precise contribution.

Methylation, a key epigenetic modification, plays a critical role in pancreatic ductal adenocarcinoma (PDAC) and other pancreatic neoplasms by regulating gene expression. Several studies have shown that aberrant methylation can lead to transcriptional silencing of tumor suppressor genes, the activation of oncogenic pathways, and changes in signaling cascades that drive pancreatic carcinogenesis. A large-scale analysis of 167 treatment-naïve, surgically resected PDACs and 29 adjacent non-tumorous pancreatic tissues revealed 11,634 CpG sites associated with 3522 genes that were differentially methylated in tumors [10]. Hypermethylation was most pronounced in the 5′ regions of genes, which included promoters and 5′UTRs. Approximately 33% of differentially methylated genes showed significant inverse correlation with mRNA expression levels, highlighting the functional impact of this epigenetic change [10]. Aberrantly methylated genes were enriched in pathways important to pancreatic cancer, including TGF-β, WNT, integrin signaling, cell adhesion, stellate cell activation, and axon guidance [10]. Within that axon guidance pathway, the SLIT-ROBO genes (SLIT2, SLIT3, ROBO1, and ROBO3) were commonly methylated, as were ITGA2 and MET, which suggests epigenetic suppression of SLIT-ROBO signaling and upregulation of MET and ITGA2 expression [10]. Notably, the hypomethylation of MET and ITGA2 correlated with higher expression and worse overall outcomes. Epigenetic inactivation of RUNX3 and the prognosis of loss of function of tumor suppressor genes via promoter hypermethylation has been described for RUNX3, an important mediator of the TGF-β signaling pathway. In one study of 32 pancreatic cancer samples, promoter hypermethylation was detected in 20 (62.5%) of these tumors and, in relation to clinicopathological data, was associated with significantly worse prognosis [11].

A study analyzed three distinct datasets of pancreatic ductal adenocarcinoma (PDAC) in two age groups: early-onset PDAC (EOPC; ≤55 years) and average-age onset PDAC (AOPC; ≥70 years). A total of 293 samples were evaluated, comprising 90 EOPC and 203 AOPC [12]. Among the known carcinogenesis-related genes, SMAD4 displayed higher mutation rates in younger patients [12]. Transcriptomic analysis indicated that the TGFβ pathway increased activation in EOPC, with higher levels of phospho-GSK3 expression [12]. These findings suggest that certain pathways, notably those involving SMAD4 and TGFβ, may distinguish younger-onset PDAC from older-onset disease. Despite evidence of distinct molecular alterations, survival outcomes revealed no differences between age groups [12]. In other words, although there may be differences in underlying biology, these did not translate into an improved or worse OS solely based on younger vs. older age at diagnosis in the analyzed datasets.

In this study, we examined the TGFB2 mRNA and *TGFB2* gene methylation as predictors of OS in PDAC. The impact of age was also examined, and the bioinformatic data were confirmed with clinical data from our PDAC trial, where patients were treated with OT-101, an antisense against TGFB2. This study uniquely demonstrates that *TGFB2* methylation and expression are prognostic factors in pancreatic ductal adenocarcinoma (PDAC) in an age-dependent manner and correlate with clinical responses to TGFB2-targeting antisense therapy. Specifically, high TGFB2 expression and low *TGFB2* promoter methylation are associated with worse overall survival in PDAC patients under the age of 65, while the use of OT-101, an antisense oligonucleotide against TGFB2, improves survival outcomes in this younger patient population. These findings highlight the isoform- and age-specific role of TGFB2 as both a biomarker and therapeutic target in PDAC.

## 2. Results

### 2.1. OT-101 P001 Clinical Trial

This Phase I/II pancreatic cancer trial (part of Protocol AP12009-P001) was designed to assess the safety, tolerability, and preliminary efficacy of trabedersen (AP 12009/OT-101) in patients with advanced (Stage IVA or IVB) pancreatic ductal adenocarcinoma. The open-label, dose-escalation structure allowed investigators to identify the MTD by incrementally increasing the daily infusion dose over a 7-day or 4-day continuous-infusion schedule. The study collected extensive safety data (including adverse events and laboratory changes), pharmacokinetic profiles, tumor response rates by imaging, and biomarkers (e.g., TGF-β2 levels and CA 19-9). Patients deriving clinical benefit could enter an extension phase for up to ten total infusion cycles. The overarching goal was to establish a safe and potentially effective recommended dose for further Phase II evaluation in PDAC.

Trabedersen (AP12009/OT-101) was systematically evaluated for safety by monitoring AEs, laboratory parameters, ECG findings, and other clinical endpoints in a dose-escalation framework. The main toxicity was thrombocytopenia, which was anticipated and generally manageable and reversible. Of the 4 patients with all-grade thrombocytopenia (out of 37 treated PDAC pts), none were considered serious and required hospitalization. Patients recovered upon stopping the investigational product, and no bleeding episodes or further complications were reported.

The clinical efficacy analysis provided encouraging signs of antitumoral activity, with overall survival in certain patient subgroups surpassing survival times currently seen with any other approved agent. For advanced-stage PDAC patients treated at a dose of 140 mg/m^2^/day (4 days-on, 10 days-off schedule), the observed overall survival was 14.5 months, which is more than double the survival time seen with approved standard treatments in second-line settings. One patient with metastatic pancreatic cancer achieved a long-term complete remission. Overall, the Phase I/II data demonstrated that trabedersen (OT-101) can induce clinically meaningful tumor responses and prolonged survival in PDAC patients. 

The clinical outcome of the trial regarding OS was integrated with the results of TCGA cohorts to establish a meaningful strategy for further clinical development, as described below.

### 2.2. TGFB2 mRNA Levels, but Not TGFB1 and TGFB3 mRNA Levels, Have a Significant Negative Prognostic Impact in Young PDAC Patients

**Figure 1:**
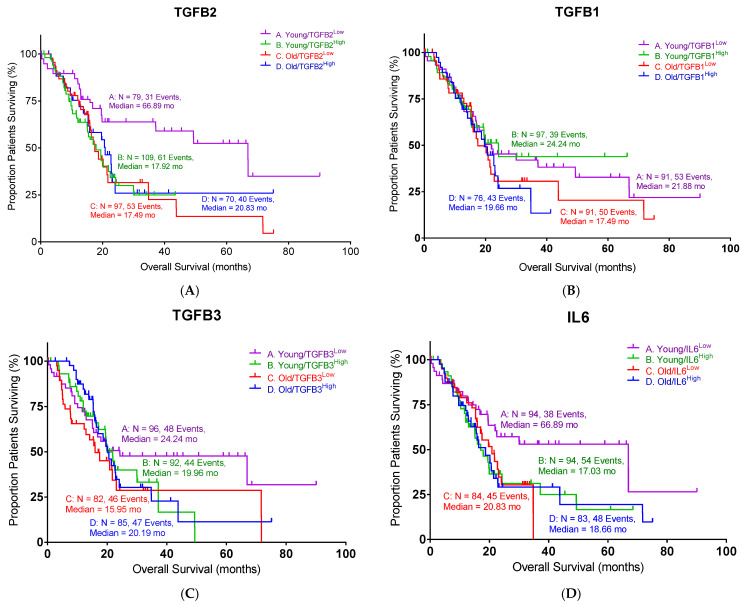
Low levels of TGFB2 and IL6 exhibited better overall survival (OS) in young PDAC patients: (**A**) Young PDAC patients with low levels of TGFB2 mRNA expression exhibited improved OS compared to patients with high levels of TGFB2 mRNA expression (log-rank *p* = 0.0006). (**B**) PDAC patients with different levels of TGFB1 mRNA expression exhibited similar OS. (**C**) PDAC patients with different levels of TGFB3 mRNA expression exhibited similar OS. (**D**) Young PDAC patients with low levels of IL6 mRNA expression exhibited improved OS compared to patients with high levels of IL6 mRNA expression (log-rank *p* = 0.0054). Events indicate death events. The *p*-values are presented as an inset in each figure (**A**–**D**), and for each figure, overall significance for the differences in the four curves was tested. Four independent statistical tests were performed for TGFB2 (**A**), TGFB1 (**B**), TGFB3 (**C**), and IL6 (**D**), and two comparisons exhibited *p*-values less than or equal to 0.0054 (false discovery rate = 0.011).

It has been demonstrated that only the TGFβ2 isoform was predictive of OS in pancreatic ductal adenocarcinoma (PDAC) [13]. We further analyzed the impact of TGF-β isoform (TGFβ1, TGFβ2, and TGFβ3) and IL6 expression on overall survival (OS) in PDAC patients (Figure 1). Patients were divided into “young” (<65 years) and “old” (≥65 years) subgroups based on the median age of the cohort to avoid selection bias and ensure balanced subgroup comparisons. Within each age subgroup, the expression of the indicated gene was further classified as “high” (H) or “low” (L) according to the median mRNA expression value. Among the three TGF-β isoforms analyzed, only TGFB2 demonstrated a statistically significant association with OS (log-rank *p* = 0.0006). Notably, the improved OS associated with lower TGFB2 expression was specific to the young subgroup, with a median OS of 66.9 months, substantially longer than the 17.9 months observed in the high-TGFB2 subgroup (Figure 1A, Table 1). Old patients showed similar median OS whether TGFB2 was high or low (20.8 vs. 17.5 months). These results suggested that TGFB2 plays an important role in young PDAC patients. In contrast, TGFB1 and TGFB3 were not significantly associated with OS (Figure 1B,C).

It has been reported that IL6 is regulated by TGF-β signaling [14,15]. Therefore, we also examined the impact of IL6 expression on the OS in the same PDAC cohort. The young PDAC patients with low IL6 levels exhibited improved median OS compared to the high-IL6 subgroup (Figure 1D, 66.9 vs. 17.0 months, log-rank *p* = 0.0054), which mirrored the findings in TGFB2. Old patients exhibited comparable median OS whether IL6 was high or low (18.7 vs. 20.8 months). Taken together, our findings suggested that a low expression of TGFB2 or IL6 is correlated with a better survival outcome in PDAC patients under 65 years of age.

### 2.3. TGFB2 Methylation Levels Have a Significant Positive Prognostic Impact in Young PDAC Patients

**Figure 2:**
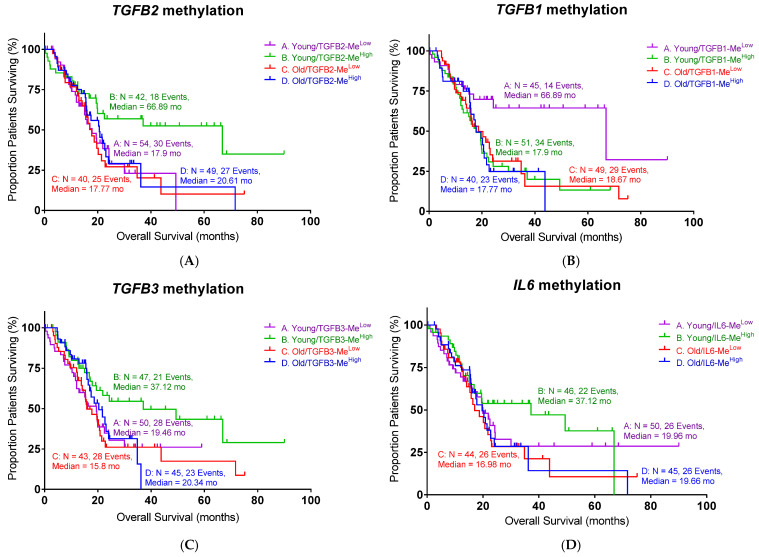
High methylation levels of *TGFB2* and low methylation levels of *TGFB1* exhibited better overall survival (OS) in young PDAC patients: (**A**) Young PDAC patients with high levels of *TGFB2* methylation exhibited improved OS compared to patients with low levels of *TGFB2* methylation (log-rank *p* = 0.0416). (**B**) PDAC patients with low levels of *TGFB1* methylation exhibited significantly improved OS (log-rank *p* = 0.0138). (**C**) PDAC patients with different levels of *TGFB3* methylation exhibited similar OS. (**D**) PDAC patients with different levels of *IL6* methylation exhibited similar OS. Events indicate death events. The *p*-values are presented as an inset in each figure (**A**–**D**), and for each figure, overall significance for the differences in the four curves was tested. Four independent statistical tests were performed for TGFB2 (**A**), TGFB1 (**B**), TGFB3 (**C**), and IL6 (**D**) methylation, and two comparisons exhibited *p*-values less than or equal to 0.0138 (false discovery rate = 0.08).

As epigenetic regulation is known to affect PDAC progression, we examined the impact of the methylation of the *TGFB1*, *TGFB2*, and *TGFB3* genes on patient survival. Among the three TGF-β isoforms, high levels of *TGFB2* methylation exhibited a significant improvement in OS in young patients, as shown in Figure 2 (log-rank *p* = 0.0416). The median survival of high *TGFB2* methylation was 66.9 months compared to the low *TGFB2* methylation group (17.9 months, Table 2). This improvement was consistent with our previous finding that lower TGFB2 expression is associated with better OS in young PDAC patients. Old patients showed no difference in OS with respect to *TGFB2* methylation levels.

Surprisingly, we found that low *TGFB1* methylation demonstrated a better OS in young PDAC patients (Figure 2B, log-rank *p* = 0.0138). The median survival of low *TGFB1* methylation in young patients was 66.9 months, whereas the median survival of high *TGFB1* methylation was 17.9 months. *TGFB3* and *IL6* methylation levels did not show significant correlations with survival. These findings demonstrated the importance of the epigenetic regulation of *TGFB* genes in young PDAC patients, particularly with *TGFB2* and *TGFB1*, suggesting that their methylation status may serve as a potential biomarker for prognosis or therapy selection.

### 2.4. TGFB2 and TGFB3 Methylation Is Negatively Correlated with mRNA Expression Levels in PDAC Patients

**Figure 3:**
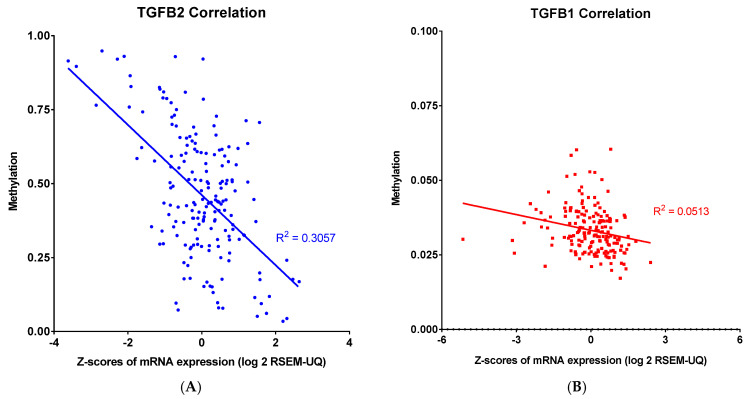

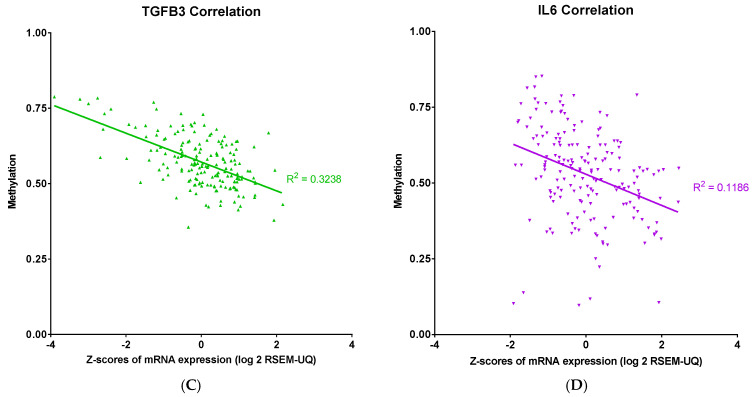
Linear correlation analysis of methylation and mRNA expression levels of TGFB2, TGFB1, TGFB3, and IL6 in PDAC. Pearson correlation analysis was performed, and each point represents an individual tumor sample, with methylation levels and mRNA abundance expressed as log2: (**A**) TGFB2 showed a robust negative correlation between methylation and mRNA expression levels in PDAC patients (R^2^ = 0.3057). (**B**) TGFB1 did not show a robust correlation between methylation and mRNA expression levels. (**C**) TGFB3 also showed a robust negative correlation between methylation and mRNA expression levels in PDAC patients (R^2^ = 0.3238). (**D**) IL6 did not show a robust correlation between methylation and mRNA expression levels. The *p*-values are presented as an inset in each figure (**A**–**D**), and for each figure, overall significance for the differences in the four curves was tested. Four independent statistical tests were performed for TGFB2 (**A**), TGFB1 (**B**), TGFB3 (**C**), and IL6 (**D**) correlations, and four comparisons exhibited *p*-values less than or equal to 0.0022 (false discovery rate = 0.002).

Our findings showed that both methylation and mRNA expression levels impacted the OS in PDAC patients. To further examine the relationship between methylation and expression levels in PDAC, we performed a linear regression to evaluate the correlation between methylation and mRNA expression for *TGFB2*, *TGFB1*, *TGFB3*, and *IL6* genes (Figure 3). There were strong correlations observed for TGFB2 (R^2^ = 0.3057, *p* < 0.0001) and TGFB3 (R^2^ = 0.3238, *p* < 0.0001), suggesting that higher promoter methylation was consistently associated with lower mRNA expression levels of these genes. In contrast, the correlations in TGFB1 (R^2^ = 0.0513, *p* = 0.0022) and IL6 (R^2^ = 0.1186, *p* < 0.0001) were much lower, with marginal deviation from the horizontal line, suggesting these genes were not modulated by this epigenetic regulation in PDAC patients.

The correlation analysis of TGFB2 and IL6 in pancreatic tumor tissues did not show a significant correlation between TGFB2 mRNA and IL6 mRNA in tumor tissues (Supplementary Figure S1A,B), despite a significant correlation in normal tissues. No correlation was observed for TGFB1 with IL6 (Supplementary Figure S1C,D). Interestingly, TGFB3 and IL6 exhibited a strong correlation in tumor tissues but not normal tissues (Supplementary Figure S1E,F). These data further point to the complexity of TGFB isoforms.

### 2.5. OS in Pancreatic Cancer Patients Treated with OT-101

**Figure 4:**
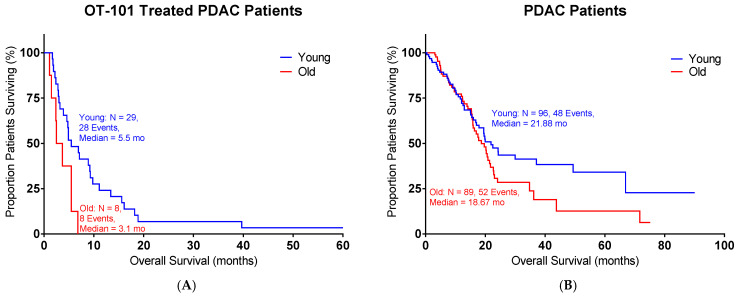
OT-101 treatment showed improved OS in young pancreatic cancer patients in a Phase I/II clinical study: (**A**) Young patients with OT-101 treatment exhibited improved OS compared to old patients with OT-101 treatment (*p* = 0.0144); median survival for the young group = 5.5 months and old group = 3.1 months. (**B**) Patients’ survival from TCGA without OT-101 treatment showed similar OS between young and old groups (*p* = 0.1509); median survival for the young group = 21.88 months and old group = 18.67 months. Age is defined as the age of diagnosis. Events indicate death events.

OT-101 (trabedersen), an 18-mer phosphorothioate antisense oligodeoxynucleotide (ASO), is designed to reduce TGFβ2 expression [16]. We examined whether OT-101 treatment could have beneficial effects in pancreatic cancer patients. A Phase I/II OT-101 multicenter dose-escalation clinical study (P001 study, Table 3) was conducted in patients with Stage III or IV with advanced pancreatic cancer (*n* = 37). Young patients treated with OT-101 showed improved OS versus old patients (Figure 4, Table 4), pushing the median OS to 5.5 months in young patients versus the 3.1 months observed in old patients (*p* = 0.0144). In contrast, the median survival in patients without OT-101 treatments did not show a significant difference between the young and old groups (*p* = 0.1509).

### 2.6. IL6 and TGFB2 Synergy in Pancreatic Cancer Patients Treated with OT-101

**Figure 5:**
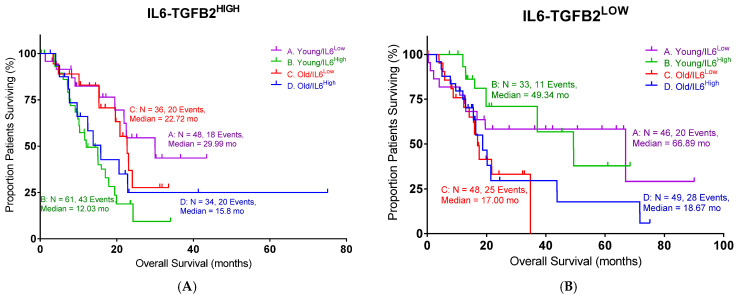

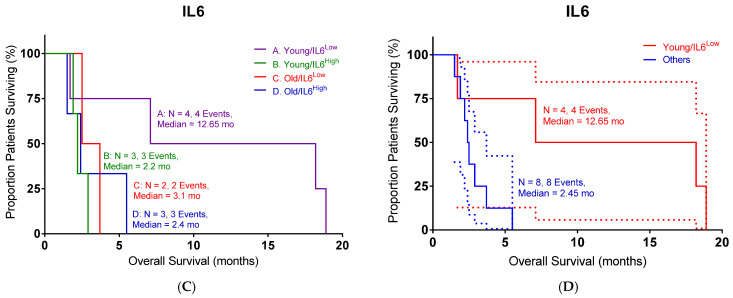
An age- and IL6-dependent separation in OS with different levels of TGFB2 in pancreatic cancer patients: (**A**) TCGA young high-TGFB2 patients with low levels of IL6 mRNA expression exhibited improved OS compared to other patients (*p* = 0.0019). (**B**) TCGA young low-TGFB2 patients with low levels of IL6 mRNA expression exhibited better OS than other patients (*p* = 0.0609). (**C**,**D**) Our clinical young patients with low levels of IL6 mRNA expression exhibited improved OS compared to other patients in OT-101-treated patients (*p* = 0.0368). The dotted lines indicate the 95% CI. Events indicate death events. The *p*-values are presented for three statistical tests, and for each figure, overall significance for the differences in the four curves was tested. Three independent statistical tests were performed, and two comparisons exhibited *p*-values less than or equal to 0.0368 (false discovery rate = 0.075).

**Figure 6:**
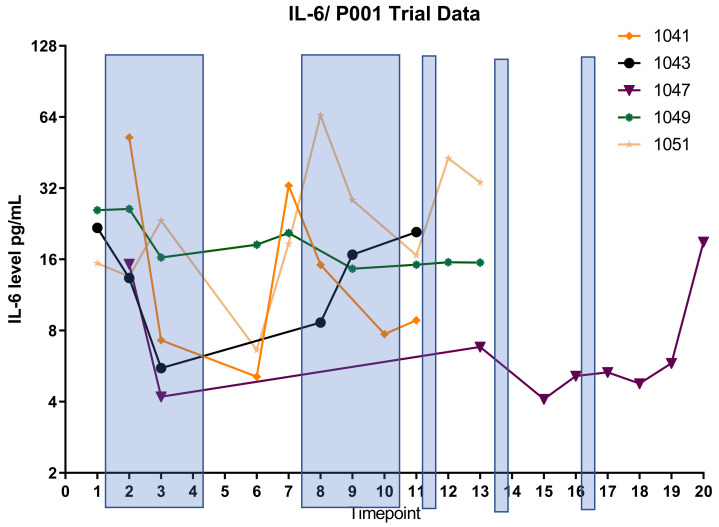
IL6 suppression by OT-101 treatment in pancreatic cancer patients. Shaded bars indicate the treatments with OT-101. Five patients exhibited a significant reduction in IL-6 levels following the first cycle of dosing with OT-101. Patients 1041 and 1051 who exhibited a rebound following treatment stop on cycle 1 exhibited a decrease again on subsequent cycle 2. All patients exhibited elevated IL-6 on progression and/or death. The definition of timepoint is in Table 7.

In order to define the potential interaction between IL6 and TGFB2, the TCGA dataset was separated into those with low TGFB2, which would correspond to OT-101 treatment, and those with high TGFB2. Patients were further split into four cohorts based on age and IL6 expression (Figure 5). In the high-TGFB2 population, we found that age had minimal impact on OS, but IL6 expression had a significant impact. Patients with low IL6 levels exhibited a better OS compared to high-IL6 groups, and this improvement showed an improved OS in young patients, with a median OS of 29.99 months (Table 5, *p* = 0.0019). In the low-TGFB2 population, age had a significant impact on OS, whereas IL6 expression did not. Improvement in OS was observed across all subgroups. A further improvement in OS was observed in the low-TGFB2 young patients with low IL6 levels (*p* = 0.0609), with a median OS of 66.89 months. These results suggested that TGFB2 can synergize with IL6 to improve OS in young pancreatic cancer patients. In our clinical study, a similar observation was made in the OT-101-treated pancreatic cancer patients versus the low-TGFB2 TCGA population. Young OT-101-treated patients with lower IL6 levels (in this case, determined as plasma IL6 levels) showed a better OS compared to other groups (*p* = 0.0368), with a median OS of 12.65 months (Table 6). IL6 levels were examined in the 12 pancreatic cancer patients from the P001 study, with OT-101 treatment at 140 mg/m^2^/day. Notably, 9 of the 12 patients examined exhibited high IL-6 levels, and more than 50% of these patients (5 of 9) exhibited a significant reduction in IL-6 levels following the first cycle of dosing with OT-101 (Figure 6, Table 7). Our results confirmed the findings in the TCGA data and demonstrated the synergistic effects of TGFB2 and IL6 in young patients.

## 3. Discussion

Our analysis underscores the critical role of TGFB2 in pancreatic ductal adenocarcinoma (PDAC) progression and prognosis, revealing significant age-dependent associations. Bioinformatic analyses from TCGA clearly demonstrated that high TGFB2 expression negatively impacted OS exclusively in younger PDAC patients (under 65 years old), significantly reducing median OS compared to patients with low expression. In contrast, TGFB1 and TGFB3 were not predictive factors. One likely explanation is that TGFB1 and TGFB3 have pleiotropic, context-dependent functions that dilute their single-agent prognostic power in PDAC. For example, TGFB1 has shown both tumor-suppressive and tumor-promoting effects depending on stage and context, leading to inconsistent correlations with patient outcomes [17,18]. In contrast, TGFB2 appears to have a more unidirectional or context-specific impact—its high expression negatively impacts overall survival, particularly in younger patients, perhaps by more directly shaping a fibrotic and immunosuppressive tumor microenvironment. Similarly, TGFB3’s expression may be elevated in PDAC [8], but its role may not independently drive aggressive tumor biology to the extent that TGFB2 does. Thus, while all three isoforms are components of the TGFβ pathway, TGFB2 may serve as a more robust predictor because its signaling appears to consistently contribute to adverse outcomes (particularly in younger PDAC patients). Both TGFB2 and IL6 appear to serve as robust predictors in PDAC because they more directly drive and reinforce a tumor microenvironment that promotes aggressive disease. For instance, elevated levels of TGFB2 are associated with a fibrotic and immunosuppressive tumor microenvironment due to its ability to foster the expansion of cancer-associated fibroblasts (CAFs) and the infiltration of tumor-associated macrophages (TAMs) [19]. In this setting, TGFB2 not only increases the deposition of extracellular matrix components (fueling fibrosis) but also helps shape an immune milieu that suppresses antitumor responses, thereby leading to worse overall survival, especially in younger patients. Similarly, IL6 is a well-known proinflammatory cytokine that can enhance tumor progression by driving persistent inflammation. IL6 can stimulate cancer cell proliferation and survival and promote the recruitment and activation of myeloid cells that further suppress antitumor immunity and facilitate tumor growth. The convergence of these pathways means that when both TGFB2 and IL6 are upregulated, their cooperative effects on the tumor stroma and the immune system are accentuated, rendering them particularly predictive of poor outcomes in PDAC.

TGF-β1 plays a pleiotropic role in cancer biology, functioning as a tumor suppressor in early carcinogenesis and a pro-tumorigenic cytokine in advanced disease through the induction of epithelial–mesenchymal transition (EMT), immune evasion, and fibrosis. In PDAC, elevated plasma TGF-β1 has been consistently linked to shortened overall survival, and dynamic changes in its levels correlate with therapeutic outcomes. In a Phase II study of galunisertib, patients experiencing on-treatment declines in TGF-β1 had improved survival, while rising levels predicted early death [20,21]. Similar associations have been observed in other malignancies. In oral and prostate cancers, high intratumoral TGF-β1 expression is associated with nodal metastasis, high Gleason score, and reduced survival [22,23]. In hepatocellular carcinoma (HCC), both plasma TGF-β1 and circulating TGF-β1-responsive regulatory T cells are independently prognostic factors for worse outcomes [24,25]. These observations underscore the clinical importance of TGF-β1 as a non-invasive biomarker of tumor progression and immune suppression in multiple cancers, including PDAC. TGF-β3 is known to substitute for TGF-β1 in T cell polarization, capable of promoting both regulatory and proinflammatory responses depending on cytokine context [26,27]. In infectious diseases such as malaria, higher levels of latent TGF-β3 correlate with milder disease, suggesting an immunomodulatory, possibly protective, role [28]. IL-6 is a key proinflammatory cytokine implicated in cancer cachexia, immune suppression, and resistance to therapy. In PDAC, elevated baseline IL-6 levels are associated with reduced survival, and in patients with concurrent high IL-10, outcomes are particularly poor [21,25]. Similar prognostic significance has been demonstrated in head and neck squamous cell carcinoma (HNSCC) and breast cancer [29,30]. IL-6 genotypes such as 174GG and 634GG correlate with increased cytokine production and have been associated with cardiovascular disease and cancer predisposition [31,32]. In inflammatory diseases, IL-6 levels also serve as independent predictors of mortality, as seen in community-acquired pneumonia [33]. While TGFB2 emerged as the most robust prognostic marker in this study, the differential behaviors of TGFB1, TGFB3, and IL6, as related to methylation, warrant further discussion. *TGFB1* promoter methylation was paradoxically associated with worse survival in younger patients, in contrast to the effect observed for TGFB2. This discrepancy may stem from TGFB1’s pleiotropic and context-dependent functions—it can act as a tumor suppressor in early PDAC stages by enforcing growth arrest, yet also promote late-stage progression via fibrosis and immune evasion. Moreover, TGFB1 expression may be governed by epigenetic mechanisms beyond promoter methylation, such as histone modifications or enhancer activity, weakening the predictive utility of methylation status alone. In contrast, TGFB3 showed no consistent association with overall survival despite demonstrating a strong inverse correlation between methylation and expression. This suggests that while TGFB3 is epigenetically regulated, its downstream effects may be redundant or compensated for by TGFB1/2 activity in PDAC. It is also possible that TGFB3’s role is more prominent in developmental or wound-healing contexts and does not independently drive aggressive tumor behavior in pancreatic cancer. Finally, IL6 expression correlated with poor outcomes only in younger patients, likely reflecting the greater impact of IL6-driven inflammation and immunosuppression in a competent immune microenvironment. In older patients, immunosenescence and baseline inflammation may obscure IL6’s effects, diminishing its prognostic value in this group.

We also identified epigenetic regulation as a key modulatory mechanism, demonstrating that increased *TGFB2* promoter methylation correlates with improved OS specifically in younger patients, presumably due to the gene silencing effects reducing TGFβ2 expression. Interestingly, an opposite methylation effect was observed for *TGFB1*, suggesting distinct epigenetic regulatory mechanisms among TGF-β isoforms and highlighting the complex interplay of methylation patterns with gene expression and clinical outcomes. A possible explanation hinges on the idea that *TGFB2* and *TGFB1* are subject to distinct epigenetic regulatory controls that lead to different functional outcomes. In the case of *TGFB2*, increased promoter methylation appears to induce gene silencing, thereby reducing its expression. This silencing likely diminishes the pro-tumorigenic effects that *TGFB2* normally exerts—such as promoting a fibrotic and immunosuppressive tumor microenvironment—which in turn correlates with improved OS in younger patients. In contrast, the opposite methylation effect observed for *TGFB1* suggests that its promoter methylation does not similarly repress gene expression. The regulation that is specific to isoforms emphasizes the intricate relationship between methylation patterns, gene expression, and clinical outcomes [34]. The observed inverse correlation between promoter methylation and mRNA expression for TGFB2 and TGFB3 suggests that these isoforms harbor DNA elements—such as well-defined CpG islands—that are particularly vulnerable to methylation-induced silencing. When methylation occurs in key regions, it may hinder the binding of transcription factors or attract methyl-binding repressor proteins, such as MeCP2, leading to the silencing of gene expression. Specific methylation patterns, such as those found between positions -666 and -426 relative to the transcription start site in the IL6 promoter, can serve as binding sites for these repressive proteins [35]. Although this study directly addressed *IL6*, a similar mechanism may be at play in *TGFB2* and *TGFB3*, contributing to their robust inverse correlations between methylation and expression. In sharp contrast, *TGFB1* and *IL6* showed weaker or absent correlations with promoter methylation. This suggests that these genes are likely governed by additional or alternate regulatory pathways that diminish the sole impact of DNA methylation. Although IL6’s promoter methylation is critical in gene silencing—as evidenced by its binding to MeCP2 and H3meK9 in non-expressing cell lines [35]—its overall expression might be modulated by potent cytokine inducers like TNF-α, which can override methylation-mediated repression under certain conditions. Thus, the strong inverse correlations for *TGFB2* and *TGFB3* suggest that their genomic regions contain methylation-sensitive elements that directly impact transcription, whereas *TGFB1* and *IL6* are under the influence of layered regulatory inputs. These insights underline the importance of considering gene-specific promoter contexts and the complexity of epigenetic regulation. By selectively modulating these distinct regulatory axes, this could open new avenues for targeted therapeutic interventions.

Clinically, our findings were validated by data from the OT-101 Phase I/II clinical trial, where the antisense oligodeoxynucleotide specifically targeting TGFB2 significantly improved OS in younger PDAC patients relative to older patients. TGFB isoforms, particularly TGFB2, play pivotal roles in PDAC progression by modulating key mechanisms such as immunosuppression, angiogenesis, metastasis, and the tumor microenvironment. Elevated levels of TGF-β2 are associated with the aggressive advancement of pancreatic cancer [36,37]. Clinical observations underscore that the dual role of TGFβ signaling—in particular, its context-dependent function as both a tumor suppressor and promoter—has complicated the efforts to target this pathway therapeutically. Trabedersen (OT-101) is an antisense oligodeoxynucleotide specifically designed to inhibit TGFB2 mRNA. Preclinical investigations have demonstrated that inhibiting TGFB2 can reduce cell proliferation and migration and reverse TGFB2-mediated immunosuppression. One study found that trabedersen diminished the secretion of TGF-β2 in human pancreatic cell lines and effectively inhibited the migration of pancreatic ductal adenocarcinoma (PDAC) cells [16]. This mode of action attenuates the fibrotic and immunosuppressive tumor microenvironment and provides a strong rationale for its use as a therapeutic agent in PDAC. Clinical findings indicate that targeting specific isoforms, particularly TGFβ2, might be more beneficial than broad TGFβ inhibition, as our results indicate that elevated levels of TGFβ2 expression adversely affected OS primarily in younger patients with PDAC. Moreover, our studies suggest that *TGFB2* and *TGFB3* may contain DNA elements particularly vulnerable to methylation-induced silencing, resulting in significant inverse correlations between promoter methylation and mRNA expression. This suggests that the biological roles of TGFB2 and TGFB3 may exhibit a more linear or unidirectional impact on disease progression. In contrast, TGFB1 and IL6 are influenced by additional regulatory factors, which may complicate the potential benefits of targeting these pathways. This isoform-specific vulnerability may contribute to the observed improved outcomes when TGFβ2 is precisely targeted. TGFB2 not only facilitates fibrosis but also fosters an immunosuppressive setting. Thus, inhibiting TGFB2 could potentially enhance T cell-mediated cytotoxicity, as evidenced by recent studies showing increased infiltration of CD8+ T cells and a reduction in regulatory T cells [38]. Conversely, in tumors where TGFβ signaling redundancy or compensatory pathways exist, the blockade of one pathway component might not be sufficient to reverse immunosuppression or tumor progression. Data from the OT-101 Phase I/II clinical trial support the targeting of TGFB2 for therapeutic purposes. The findings indicate that an antisense oligodeoxynucleotide specifically aimed at TGFB2 notably enhanced overall survival in younger patients with pancreatic ductal adenocarcinoma when compared to older patients. This suggests that certain patient subgroups, potentially characterized by unique epigenetic or microenvironmental factors, may experience greater benefits from TGFB2 inhibition. Coupled with encouraging preclinical results—such as the inhibition of cell proliferation, migration, and the reversal of immunosuppression—alongside positive clinical outcomes in younger individuals, OT-101 shows promise as a component of combination treatment strategies for PDAC. However, the necessity to further elucidate which patients are most likely to respond based on molecular and epigenetic tumor profiles remains, thereby optimizing the use of OT-101 in a precision medicine approach.

The age-related differences in TGFB2 expression and methylation may reflect a shift in the tumor microenvironment and immune response, particularly in the context of T cell engagement and mesenchymal–epithelial transition. The hypermethylation of *TGFB2* is associated with lower TGFB2 expression and improved patient survival. Importantly, multivariate analysis shows that this phenomenon is independent of SMAD4 and canonical TGFβ signaling, suggesting a non-SMAD, immune-related role for TGFB2 methylation [39]. Additionally, *TGFB2* methylation correlates inversely with numerous T cell receptor (TCR) signaling components—HLA-D molecules, CD3D, CD28, and LCK—all of which are upregulated in the tumor when *TGFB2* is weakly methylated [39]. This suggests that low methylation (high TGFB2 expression) may suppress T cell engagement and undermine a robust adaptive immune response, while high methylation (low TGFB2) may allow greater T cell infiltration and activity. Finally, the expression of TGFB2 is associated with the genes involved in mesenchymal–epithelial transition [40]. Tumors with low *TGFB2* methylation may be more prone to a mesenchymal phenotype, which typically correlates with an immunosuppressive microenvironment and reduced T cell infiltration. Age-related epigenomic alterations might influence this process by adding methyl groups to *TGFB2*, thereby dampening its expression and shifting the tumor toward a more “epithelial-like” and less immunosuppressive state [41]. Clinically, higher *TGFB2* methylation corresponds to a more robust T cell-mediated immune response and a less mesenchymal, less aggressive tumor, contributing to improved patient survival. Furthermore, this profile may help identify patients who might respond to immunotherapy (such as T cell receptor therapies or checkpoint inhibitors) and MET-targeted therapies (like trametinib or CAR-M-c-MET) [42,43]. Aging leads to significant changes in the immune system, a phenomenon referred to as immunosenescence. This process is characterized by several key features, including the involution of the thymus, which restricts the production of new naïve T cells, an imbalance between naïve and memory T cells, altered metabolic processes, and extensive epigenetic changes. The disruption of T cell populations, along with persistent antigen stimulation, contributes to the early senescence of immune cells. As these senescent immune cells age, they develop a proinflammatory secretory phenotype that intensifies the phenomenon known as inflammaging [44]. This overall decline results in a reduced capacity to mount antitumor immune responses, which is why older patients often experience a diminished benefit from therapies that rely on a robust immune system. Several studies provide evidence for these age-related alterations. The chronic inflammatory state or inflammaging observed in older individuals further blunts the immune rescue mechanisms that might otherwise compensate for the tumor’s escape signals. Given the documented decline in immune competence with age, it becomes critical to tailor immunotherapeutic strategies for older patients. The idea behind age-specific dosing and immuno-priming is that reversing or lessening immune senescence could improve the effectiveness of cancer treatments. Younger patients, who retain a more effective pool of cytotoxic T and NK cells, can capitalize on the uncloaked tumor environment following TGFB2 blockade. In contrast, older patients—with their age-related immune deficits—may gain incremental benefit from a dual strategy where TGFB2 inhibition is paired with immuno-priming to overcome the blunted immune response. This dual approach aims to excise key immunosuppressive nodes while simultaneously boosting the residual immune function in older hosts. The successful implementation of age-specific dosing or immuno-priming requires well-designed, biomarker-guided clinical studies. For example, evaluating dynamic pharmacodynamic readouts such as IL-6 or phospho-STAT3 levels will help determine the optimal timing and dosing of these interventions in conjunction with TGFB2 blockade. The identification of age-related epigenetic profiles may further allow for the rational combination of agents (e.g., DNMT inhibitors for hypermethylation of TGFB2 loci) with immuno-priming therapies to enhance treatment durability.

This study has some notable limitations and therefore primarily generates a hypothesis that should be confirmed with additional studies to be performed in the future, primarily due to the use of bioinformatics analyses of the TCGA dataset. Additional laboratory confirmation of TGFB1, TGFB2, TGFB3, and IL6 gene methylation and gene expression would be needed for further validation. The validation of the mRNA markers will require techniques such as quantitative RT-PCR and immunohistochemistry. It is possible that these differences are reflective of age-dependent changes in immunity; therefore, we are actively looking at identifying immune cells involved in the tumor microenvironment responsible for these observations. We attempted to validate the TCGA analyses using data from our Phase I/II PDAC trial for OT-101, a suppressor of TGFB2. A number of limitations should be pointed out for our clinical study, including its heterogeneous nature, single-arm design, and small size for the patient population. It will be important to further evaluate the clinical potential of OT101 in a larger and more homogenous patient population in our current Phase III PDAC trial, OT-01-P201 (NCT06079346). OT-01-P201 is designed as a randomized, open-label, active-controlled, multicenter Phase IIB/III study designed to compare the efficacy and safety of OT-101 in combination with modified FOLFIRINOX (folinic acid, 5-FU, irinotecan, and oxaliplatin) to modified FOLFIRINOX alone in patients with advanced and unresectable or metastatic pancreatic cancer.

## 4. Materials and Methods

### 4.1. Domain-Specific Identification of PubMed Articles Augmented by Artificial Intelligence

After searching PubMed using appropriate keywords (aging, cancer, il6, tgfbeta, methylation, and pancreatic cancer), the resulting 19,510 abstracts were downloaded as text documents for processing using the Chatbot-enabled tools developed at Oncotelic Therapeutics (Agoura Hills, CA, USA). Each abstract was then (aided using puppeteer 19.11.1) embedded and transformed (langchain-openai 0.2.3, openai 1.52.0) into a vector of numbers capturing semantic similarity between text elements (tokens) and then stored in our Qdrant vector database (https://qdrant.tech/). Semantically similar abstracts were transformed into the same vector “embedding” space, as the embedding model was trained to minimize the distance between pairs of abstracts in this space. Using an agglomerative clustering algorithm (hdbscan 0.8.39) to group the vectors, we automatically labeled these clusters using the question-answering model to identify any similarity between the abstracts corresponding to each cluster’s vectors. During the question-answering process, the user’s query was converted into an embedding vector. A similarity metric, such as cosine similarity, was then utilized to find the embedded abstract vectors nearest to the vector representing the query. The abstracts that corresponded to the nearest vectors were subsequently provided to the question-answering model as context, alongside the original query, to produce a response to the user’s question.

The React framework served as the backbone of the user interface, providing an open source and flexible solution for developing powerful front-end user interfaces (https://react.dev/, accessed on 25 March 2024). In addition, we used the @mui/material (https://mui.com/, accessed on 25 March 2024) libraries for the interface’s design aspects and aimed to follow the material design guidelines closely. Serving the front end was Node.Js (https://nodejs.org/en, accessed on 25 March 2024). The Node.js libraries included in the project were @adobe/pdfservices-node-sdk 3.4.2@aws-sdk/client-s3 3.412.0, @langchain/community 0.2.5, @material-ui/core 4.12.4, @mui/base 5.0.0-beta.18, @mui/icons-material 5.11.16, @mui/material 5.15.20, @mui/styled-engine-sc 5.12.0, @mui/x-date-pickers 6.15.0, @qdrant/js-client-rest 1.4.0, carrot2 0.0.1, pdf2img 0.5.0, pdfjs-dist 4.5.136, puppeteer 19.11.1, react 18.2.0, sequelize 6.31.1, and zod 3.22.4.

The integration of our Chatbot technology applied to PubMed abstracts facilitated the rapid discovery of key primary publications used in the writing of this manuscript.

### 4.2. TCGA and TNM Data

Clinical and transcriptomic data of pancreatic ductal adenocarcinoma (PDAC) patients were obtained from The Cancer Genome Atlas (TCGA) database via the cBioPortal web analysis portal (https://www.cbioportal.org/study/summary?id=paad_icgc%2Cpaad_qcmg_uq_2016%2Cpaad_tcga%2Cpaad_tcga_pan_can_atlas_2018%2Cpaad_utsw_2015%2Cpaad_cptac_2021, accessed on 13 April 2025). The dataset used in this study corresponds to the TCGA-PAAD (Pancreatic Adenocarcinoma) project to determine the prognostic impact of TGFB1, TGFB2, TGFB3, and IL6 median expression cut-off values for high versus low patient sub-groupings on OS for all PDAC patients (*n* = 355) and PDAC patient with different methylation levels (*n* = 185). Data were downloaded in accordance with TCGA guidelines and usage policies. The data portal offers the option to view gene expression as Z-scores for transcripts per million (TPM), calculated using RNA-Seq with the expectation-maximization (RSEM) algorithm [45]. Briefly, RSEM normalizes TPM through a three-step process. It first aligns RNA-Seq reads to a reference transcriptome using a probabilistic expectation-maximization (EM) algorithm to estimate the origins of multi-mapping reads. Next, it calculates the expected number of fragments per transcript, considering transcript length and sequencing depth. Finally, RSEM computes reads per kilobase (RPK) for each transcript and scales these values to achieve a total of one million, yielding TPM = (RPK/sum RPKs) × 10^6^. The TPM values are then log2-transformed.

The mRNA expression levels of target genes and correlation analysis in pancreatic ductal adenocarcinoma (PDAC) tumor and normal tissues were analyzed using the TNMplot public database (https://tnmplot.com/analysis/, accessed on 13 April 2025).

The gene expression data normalization method for RNA-Seq data in TNMplot involves a two-step process. In the first step, DESeq2 algorithm employs the median-of-ratios method to normalize raw aligned HTSeq-counts by estimating size factors; it computes the geometric mean of counts for each gene across all samples, calculates the ratio of each gene’s count to this geometric mean for each sample, and determines the size factor as the median of these ratios. Raw counts are then divided by their respective size factors, thus correcting for variations in sequencing depth and RNA composition. The second step typically involves an additional scaling normalization to further harmonize the data, especially when merging datasets across multiple sources, such as cancers for TCGA and normal tissue counts from GTEx [46].

In cBioPortal, methylation levels are represented as beta-values, which range from 0 (indicating unmethylated) to 1 (indicating fully methylated), related to the proportion of methylated signal intensity relative to the total signal (methylated and unmethylated). To account for the presence of multiple probes associated with a single gene, cBioPortal selects a representative probe, typically the one most anti-correlated with gene expression.

Survival analyses and linear correlation analyses were performed using GraphPad Prism version 6.7 for Windows (Boston, MA, USA).

### 4.3. OS Outcomes and IL6 Levels for PDAC Patients Treated with OT-101

Our clinical trial (NCT00844064) was approved by the Ethics Committee of the Charité Berlin Campus VirchowKlinikum (Berlin, Germany). Written informed consent was obtained from all study subjects, and all participating centers provided ethical committee approval.

The Phase I/II trial of trabedersen (AP 12009/OT-101) for pancreatic ductal adenocarcinoma (PDAC) was designed as an open-label, multi-center dose-escalation study to assess the safety and tolerability of OT-101 in adult patients with TGF-β2 overproducing advanced solid tumors. Age at diagnosis was used, thus avoiding confounding factors such as prior chemotherapy, radiotherapy, or surgical interventions. The study incorporated a cohort-based dose-escalation strategy, enrolling 3 to 6 patients per cohort at increasing dose levels until the maximum tolerated dose (MTD) was determined. Upon reaching the MTD, additional patients were treated at the recommended Phase II dose. The trial featured both initial (7-day continuous infusion every 2 weeks) and modified (4-day continuous infusion every 2 weeks) schedules, with dose levels up to 480 mg/m^2^/day and 590 mg/m^2^/day, respectively. The study consisted of a core period involving two full cycles of therapy, with the option for patients showing clinical benefit or stable disease to receive up to eight additional extension cycles, totaling ten cycles. Follow-up visits were scheduled every 8 weeks post-infusion, regardless of the number of cycles completed. Inclusion criteria required patients to be between 18 and 75 years old, with histologically confirmed Stage IVA or IVB pancreatic cancer, a Karnofsky performance status of at least 80%, and adequate organ function. Exclusion criteria included inability to comply with protocol; active infections such as HIV, HBV, or HCV; significant cardiovascular issues; brain metastases; and recent antitumor therapies. The primary endpoint was the determination of the MTD, while secondary endpoints involved safety assessments, cardiac monitoring, tumor response, overall survival, TGF-β2 plasma concentrations, and pharmacokinetic profiling of AP 12009. Comprehensive safety assessments were maintained throughout the study, including vital signs, physical exams, ECGs, laboratory tests, and adverse event monitoring. Tumor assessments were conducted via CT scans every 8 weeks to evaluate response categories such as complete response, partial response, stable disease, and progressive disease. Biomarker analyses included evaluating AP 12009 pharmacokinetics and TGF-β2 levels, as well as tumor markers CA 19-9 and CEA, which are crucial indicators in advanced PDAC.

Safety assessments in this study encompassed adverse events (AEs), laboratory analyses (hematology, biochemistry, and urinalysis), electrocardiograms (ECGs), cardiac monitoring, physical examinations, vital signs, and performance status measurements. All adverse events (AEs) were listed using coding for System Organ Class (SOC) and Preferred Term (PT) (using the MedDRA version 9.0 or later). Treatment-emergent AEs (TEAEs) were defined as any AEs occurring or worsening after the start (date and time) of the first infusion and before 28 days after the last treatment (stop day of last infusion, including extension cycles). Moreover, AEs occurring or worsening later than 4 weeks after the last treatment were also defined as treatment-emergent if these were considered to be related to the study drug. Dose-limiting toxicities (DLTs) and unacceptable toxicities were captured during the core study period (two cycles) and, if applicable, during extension cycles. These events were exhaustively tracked to guide dose-escalation decisions and ensure patient safety. DLTs or unacceptable toxicities were summarized by cycle and by schedule, cohort, and indication, for final- and recommended-dose cohorts. Investigators also recorded action taken with respect to the investigational product and other interventions as needed. Each AE was classified by system organ class (SOC) and preferred term (PT), with additional analysis by severity (graded using NCI-CTC grades or 4-point-severity scale if NCI-CTC grades were not available), relationship to trial medication, action taken (e.g., dose interrupted, dose reduced, study drug withdrawn), outcome, and seriousness. Laboratory data included hematology, biochemistry, and urinalysis. Investigators flagged out-of-range values as clinically relevant (clin. rel.) or non-clinically relevant (non-clin. rel.). Laboratory values were converted to SI units, and reference ranges were standardized to facilitate comparability across sites. For hematology and serum chemistry, NCI-CTC grades are reported for those hematology and biochemistry parameters where NCI-CTC grading is available using NCI-CTC Lab criteria (NCI-CTC v2.0).

Efficacy was primarily assessed via imaging-based tumor response (RECIST criteria) with computed tomography (CT) scans performed every 8 weeks, as well as through time-related endpoints such as time to progression (TTP), progression-free survival (PFS), and overall survival (OS). The results of tumor assessments were analyzed in the evaluable efficacy population. Tumor markers (e.g., CA 19-9 and/or CEA) were also measured in patients with pancreatic or colorectal cancer.

The study used RECIST-based categories of complete response (CR), partial response (PR), stable disease (SD), and progressive disease (PD). An additional category of unknown (UK) was applied if no definitive assessment could be made. For each 8-week interval, investigators evaluated the overall best response; rates of CR, PR, SD, and PD were summarized. The “best overall tumor response” referred to the best response the patient achieved at any of the scheduled assessments. Response-related endpoints included the complete response rate (CRR), the partial response rate (PRR), the overall response rate (CR + PR), and the tumor growth control rate (CR + PR + SD). Time to progression and PFS were analyzed using Kaplan–Meier methods, with summaries including median estimates in days, weeks, months, and 95% confidence intervals. Overall survival was similarly evaluated by Kaplan–Meier analysis. Non-deceased or lost-to-follow-up patients were censored at the last date known to be alive.

The following 31 analytes were measured: EGF, eotaxin, FGF-basic, G-CSF, granulocyte–monocyte colony-stimulating factor, HGF, IFN-α, IFN-γ, IL-1RA, IL-1β, IL-2, IL-2R, IL-4, IL-5, IL-6, IL-7, IL-8, IL-10, IL-12 (p40/p70), IL-13, IL-15, IL-17, IP-10, MCP-1, MIG, MIP-1α, MIP-1β, RANTES, TNF-α, VEGF, and MICA. Cyto-/chemokine concentration in pg/mL for each plasma sample was measured in duplicate. In cases where the concentration was too low to be determined, the values of the detection limit were used for further calculations. Detection limits were provided by the manufacturer of the assay (Lophius Biosciences). In cases where the concentration was too high to be determined, the 1.5-fold value of the highest standard for the applicable cyto-/chemokine that was measured on the plate was used for further calculations. Plasma levels of IL6 were tracked over three cycles of OT-101 therapy (140 mg/m^2^/day) in 12 PDAC patients. Samples were acquired before the onset of OT-101 therapy and at eight selected time points during therapy.

## 5. Conclusions

Our study highlights the isoform-specific and age-dependent prognostic significance of TGFB2 in pancreatic ductal adenocarcinoma (PDAC). Among the three TGF-β isoforms, only TGFB2 mRNA expression was significantly associated with overall survival (OS), particularly in patients under 65 years of age. Young PDAC patients with low TGFB2 expression exhibited a significantly improved median OS compared to those with high TGFB2 levels, whereas this improved OS was not observed in older patients. Consistently, higher *TGFB2* promoter methylation, often associated with gene silencing, also correlated with longer OS in young patients. These findings demonstrate that reduced TGFB2 expression confers a favorable prognosis in PDAC patients. Furthermore, IL6, a downstream effector of TGF-β signaling, followed a similar pattern in patient survival, suggesting a potential mechanistic link between TGFB2 activity and pro-tumorigenic cytokine signaling in younger PDAC patients. Notably, we identified strong negative correlations between the methylation levels of *TGFB2* and *TGFB3* versus their mRNA expression levels, respectively, indicating that epigenetic regulation plays a critical role in modulating gene expression and disease outcome. Importantly, our clinical evaluation of OT-101 (trabedersen), a TGFB2-targeting antisense oligonucleotide, demonstrated improved survival in young PDAC patients, unveiling the therapeutic potential of isoform-specific TGF-β inhibition. Taken together, these findings support TGFB2 as both a prognostic biomarker and a promising therapeutic target, particularly in younger PDAC patients, and reveal further possibilities for exploration of epigenetic and transcriptomic biomarkers to provide personalized treatment strategies in this challenging disease.

## Figures and Tables

**Table 1:** The statistical summary of PDAC patients in Figure 1.

Gene	Young/Low	Young High	Old/Low	Old/High	Log-Rank *p*
TGFB2	66.89	17.92	17.49	20.83	0.0006
TGFB1	21.88	24.24	17.49	19.66	n.s.
TGFB3	24.24	19.96	15.95	20.19	n.s.
IL6	66.89	17.03	20.83	18.66	0.0054

Age is defined as age at diagnosis. The n.s. indicates no significant differences.

**Table 2:** The statistical summary of PDAC patients in Figure 2.

Gene	Young/Low	Young/High	Old/Low	Old/High	Log-Rank *p*
TGFB2	17.90	66.89	17.77	20.61	0.0416
TGFB1	66.89	17.90	18.67	17.77	0.0138
TGFB3	19.46	37.12	15.80	20.34	n.s.
IL6	19.96	37.12	16.98	19.66	n.s.

Age is defined as age at diagnosis. The n.s. indicates no significant differences.

**Table 3:** The demographic table for the pancreatic cancer patients in our clinical trial.

Characteristic	Pancreatic Cancer (*n* = 37)
Age (years)	
Median (Range)	63 (40–76)
Gender	
Male	17 (46%)
Female	20 (54%)
Race (*n*, %)	
Caucasian	37 (100.0)
Weight (kg)	
Median (Q1, Q3)	64.0 (55.0, 73.0)
Height (cm)	
Median (Q1, Q3)	169.5 (163.0, 175.0)
Tumor Staging (AJCC)	
Stage III	4 (11%)
Stage IV	33 (89%)
Unknown	**0**
Median Overall Survival (months)	4.9
Previous Therapies (median)	
Total	2
Chemotherapy	2
Immunotherapy	0
CA-19 Baseline (U/mL)	
Median	960.5
Range	6–236,040
CA 19-9 End of Treatment (U/mL)	
Median	3021
Range	2–582,084
CEA Baseline (µg/L)	
Median	11.5
Range	1–752
CEA End of Treatment (µg/L)	
Median	32.2
Range	1–1668
Overall Tumor response	
Tumor growth control	15 (41.7%)
Any response	1 (2.8%)
- Complete response	1 (2.8%)
- Partial response	0
Stable disease	14 (38.9%)

**Table 4:** The demographic table for the OT-101-treated subgroups in pancreatic cancer patients in our clinical trial.

Characteristic	Young (*n* = 29)	Old (*n* = 8)	Total (*n* = 37)
OS (months), median (range)	5.5 (1.7–76.2)	3.1 (1.1–6.8)	4.9 (1.1–76.2)
Age, median (range), years	61 (40–67)	72.5 (67–76)	63 (40–76)
Male	15 (51.7%)	3 (37.5%)	18 (48.6%)
Female	14 (48.3%)	5 (62.5%)	19 (51.4%)
Tumor staging (AJCC)—Stage III	3 (10.3%)	1 (12.5%)	4 (10.8%)
Tumor staging (AJCC)—Stage IV	26 (89.7%)	7 (87.5%)	33 (89.2%)
Histology grading 1	3 (10.3%)	0 (0%)	3 (8.1%)
Histology grading 2	11 (37.9%)	5 (62.5%)	16 (43.2%)
Histology grading 3	10 (34.5%)	2 (25%)	12 (32.4%)
Histology grading unknown	5 (17.2%)	1 (12.5%)	6 (16.2%)

Age is defined as age at treatment.

**Table 5:** The statistical summary of pancreatic cancer patients in Figure 5.

Group	Young/IL6^Low^	Young/IL6^High^	Old/IL6^Low^	Old/IL6^High^	Log-Rank *p*
TGFB2^High^	29.99	12.03	22.72	15.8	0.0019
TGFB2^Low^	66.89	49.34	17	18.67	0.0609
OT-101	12.65	2.2	3.1	2.4	0.0368

Age is defined as age at diagnosis.

**Table 6:** The demographic table for the IL6 subgroups in pancreatic cancer patients in our clinical trial.

Characteristic	Young/IL6^Low^ (*n* = 4)	All Others (*n* = 8)	Total (*n* = 12)
OS (months), median (range)	12.65 (1.7–18.9)	2.45 (1.5–5.5)	2.70 (1.5–18.9)
Age, median (range), years	63.5 (43–65)	68.5 (40–75)	64.5 (40–75)
Male	2 (50%)	1 (12.5%)	3 (25%)
Female	2 (50%)	7 (87.5%)	9 (75%)
Tumor staging (AJCC)—Stage III	1 (25%)	0 (0%)	1 (8.3%)
Tumor staging (AJCC)—Stage IV	3 (75%)	8 (100%)	11 (91.7%)

Age is defined as age at treatment.

**Table 7:** The definition of timepoint in Figure 6.

Timepoint	Study Phase	Timepoint	Study Phase	Timepoint	Study Phase
1	Screening|prior to start	10	Ext. Period Cycle 3|Day 1, prior start	18	FU visit|FU 2
2	Cycle 1|Day 2	11	Ext. Period Cycle 3|Day 5, after stop	19	FU visit|FU 3
3	Cycle 1|Day 5	12	Ext. Period Cycle 4|Day 1, prior start	20	FU visit|FU 4
6	Cycle 2|Day 15, prior to start	13	Ext. Period Cycle 4|Day 5, after stop	21	FU visit|FU 5
7	Cycle 2|Day 16	15	Ext. Period Cycle 5|Day 1, prior start	22	FU visit|FU 6
8	Cycle 2|Day 19	16	Ext. Period Cycle 5|Day 5, after stop	23	FU visit|FU 7
9	Final visit|Day 29	17	FU visit|FU 1		

## Data Availability

We utilized The Cancer Genome Atlas (TCGA) database via the Genomic Data Commons (GDC) Data Portal (https://portal.gdc.cancer.gov/, accessed on 13 April 2025). We analyzed the mRNA expression levels of target genes and correlation analysis in pancreatic ductal adenocarcinoma (PDAC) tumor and normal tissues using the TNMplot public database (https://tnmplot.com/analysis/, accessed on 13 April 2025).

## Supplementary Materials

The following supporting information can be downloaded at: https://www.mdpi.com/article/10.3390/ijms26136357/s1.

## References

[1] Truty M.J., Urrutia R. (2007). Basics of TGF-beta and pancreatic cancer. Pancreatology.

[2] Lin X., Feng X.H. (2005). Abrogation of transforming growth factor-beta signaling in pancreatic cancer. World J. Surg..

[3] Ijichi H. (2004). TGF-beta signaling pathway in pancreatic cancer cells. Nihon Rinsho.

[4] Ito D., Fujimoto K., Doi R., Koizumi M., Toyoda E., Mori T., Kami K., Kawaguchi Y., Whitehead R., Imamura M. (2004). Chronic exposure of transforming growth factor beta 1 confers a more aggressive tumor phenotype through downregulation of p21(WAF1/CIP1) in conditionally immortalized pancreatic epithelial cells. Surgery.

[5] Shen W., Tao G.Q., Zhang Y., Cai B., Sun J., Tian Z.Q. (2017). TGF-beta in pancreatic cancer initiation and progression: Two sides of the same coin. Cell Biosci..

[6] Principe D.R., DeCant B., Mascarinas E., Wayne E.A., Diaz A.M., Akagi N., Hwang R., Pasche B., Dawson D.W., Fang D. (2016). TGFbeta Signaling in the Pancreatic Tumor Microenvironment Promotes Fibrosis and Immune Evasion to Facilitate Tumorigenesis. Cancer Res..

[7] Hilbig A., Oettle H. (2011). Transforming growth factor beta in pancreatic cancer. Curr. Pharm. Biotechnol..

[8] Friess H., Yamanaka Y., Buchler M., Ebert M., Beger H.G., Gold L.I., Korc M. (1993). Enhanced expression of transforming growth factor beta isoforms in pancreatic cancer correlates with decreased survival. Gastroenterology.

[9] Fukumura Y., Kumasaka T., Mitani K., Karita K., Suda K. (2006). Expression of transforming growth factor beta1, beta2, and beta3 in chronic, cancer-associated, obstructive pancreatitis. Arch. Pathol. Lab. Med..

[10] Nones K., Waddell N., Song S., Patch A.M., Miller D., Johns A., Wu J., Kassahn K.S., Wood D., Bailey P. (2014). Genome-wide DNA methylation patterns in pancreatic ductal adenocarcinoma reveal epigenetic deregulation of SLIT-ROBO, ITGA2 and MET signaling. Int. J. Cancer.

[11] Nomoto S., Kinoshita T., Mori T., Kato K., Sugimoto H., Kanazumi N., Takeda S., Nakao A. (2008). Adverse prognosis of epigenetic inactivation in RUNX3 gene at 1p36 in human pancreatic cancer. Br. J. Cancer.

[12] Ben-Aharon I., Elkabets M., Pelossof R., Yu K.H., Iacubuzio-Donahue C.A., Leach S.D., Lowery M.A., Goodman K.A., O’Reilly E.M. (2019). Genomic Landscape of Pancreatic Adenocarcinoma in Younger versus Older Patients: Does Age Matter?. Clin. Cancer Res..

[13] Qazi S., Trieu V. (2024). TGFB2 mRNA Levels Prognostically Interact with Interferon-Alpha Receptor Activation of IRF9 and IFI27, and an Immune Checkpoint LGALS9 to Impact Overall Survival in Pancreatic Ductal Adenocarcinoma. Int. J. Mol. Sci..

[14] Xue V.W., Chung J.Y., Cordoba C.A.G., Cheung A.H., Kang W., Lam E.W., Leung K.T., To K.F., Lan H.Y., Tang P.M. (2020). Transforming Growth Factor-beta: A Multifunctional Regulator of Cancer Immunity. Cancers.

[15] Huang H., Zhang Y., Gallegos V., Sorrelle N., Zaid M.M., Toombs J., Du W., Wright S., Hagopian M., Wang Z. (2019). Targeting TGFbetaR2-mutant tumors exposes vulnerabilities to stromal TGFbeta blockade in pancreatic cancer. EMBO Mol. Med..

[16] Schlingensiepen K.H., Jaschinski F., Lang S.A., Moser C., Geissler E.K., Schlitt H.J., Kielmanowicz M., Schneider A. (2011). Transforming growth factor-beta 2 gene silencing with trabedersen (AP 12009) in pancreatic cancer. Cancer Sci..

[17] Glazer E.S., Welsh E., Pimiento J.M., Teer J.K., Malafa M.P. (2017). TGFbeta1 overexpression is associated with improved survival and low tumor cell proliferation in patients with early-stage pancreatic ductal adenocarcinoma. Oncotarget.

[18] Zhao J., Liang Y., Yin Q., Liu S., Wang Q., Tang Y., Cao C. (2016). Clinical and prognostic significance of serum transforming growth factor-beta1 levels in patients with pancreatic ductal adenocarcinoma. Braz. J. Med. Biol. Res..

[19] Yu X., Chen X., Chen W., Han X., Xie Q., Geng D., Guo G., Zhou L., Tang S., Chen J. (2024). TGFbeta2 Promotes the Construction of Fibrotic and Immunosuppressive Tumor Microenvironment in Pancreatic Adenocarcinoma: A Comprehensive Analysis. Mol. Biotechnol..

[20] Javle M., Li Y., Tan D., Dong X., Chang P., Kar S., Li D. (2014). Biomarkers of TGF-beta signaling pathway and prognosis of pancreatic cancer. PLoS ONE.

[21] Giannelli G., Mikulits W., Dooley S., Fabregat I., Moustakas A., ten Dijke P., Portincasa P., Winter P., Janssen R., Leporatti S. (2016). The rationale for targeting TGF-beta in chronic liver diseases. Eur. J. Clin. Invest..

[22] Chen M.F., Wang W.H., Lin P.Y., Lee K.D., Chen W.C. (2012). Significance of the TGF-beta1/IL-6 axis in oral cancer. Clin. Sci..

[23] Yan Y., Zhang J., Li J.H., Liu X., Wang J.Z., Qu H.Y., Wang J.S., Duan X.Y. (2016). High tumor-associated macrophages infiltration is associated with poor prognosis and may contribute to the phenomenon of epithelial-mesenchymal transition in gastric cancer. OncoTargets Ther..

[24] An Y., Gao S., Zhao W.C., Qiu B.A., Xia N.X., Zhang P.J., Fan Z.P. (2018). Transforming growth factor-beta and peripheral regulatory cells are negatively correlated with the overall survival of hepatocellular carcinoma. World J. Gastroenterol..

[25] Park S.J., Park J.Y., Shin K., Hong T.H., Kim Y., Kim I.H., Lee M. (2024). The Role of Pretreatment Serum Interleukin 6 in Predicting Short-Term Mortality in Patients with Advanced Pancreatic Cancer. Biomedicines.

[26] Wang J., Zhao X., Wan Y.Y. (2023). Intricacies of TGF-beta signaling in Treg and Th17 cell biology. Cell Mol. Immunol..

[27] Veldhoen M., Hocking R.J., Atkins C.J., Locksley R.M., Stockinger B. (2006). TGFbeta in the context of an inflammatory cytokine milieu supports de novo differentiation of IL-17-producing T cells. Immunity.

[28] Ndoricyimpaye E.L., Van Snick J., Niyoyita J.D., Kanimba P., Mbonimpa J.B., Rutayisire R., Rutayisire R., Ndahindwa V., Cheou P., Coutelier J.P. (2022). Integrated Analysis of Cytokine Profiles in Malaria Patients Discloses Selective Upregulation of TGF-beta1, beta3, and IL-9 in Mild Clinical Presentation. Int. J. Mol. Sci..

[29] Merlano M.C., Denaro N., Galizia D., Abbona A., Paccagnella M., Minei S., Garrone O., Bossi P. (2023). Why Oncologists Should Feel Directly Involved in Persuading Patients with Head and Neck Cancer to Quit Smoking. Oncology.

[30] Ma Y., Ren Y., Dai Z.J., Wu C.J., Ji Y.H., Xu J. (2017). IL-6, IL-8 and TNF-alpha levels correlate with disease stage in breast cancer patients. Adv. Clin. Exp. Med..

[31] Rostamzadeh Khameneh Z., Mohammadian M., Eishi Oskuie A., Asghari R., Nemati M. (2023). Evaluation the -174G>C Genetic Polymorphism of Interleukin-6 in Iranian Patients with Chronic Lymphocytic Leukemia. Iran. J. Pathol..

[32] Panoulas V.F., Douglas K.M., Smith J.P., Stavropoulos-Kalinoglou A., Metsios G.S., Nightingale P., Kitas G.D. (2009). Transforming growth factor-beta1 869T/C, but not interleukin-6 -174G/C, polymorphism associates with hypertension in rheumatoid arthritis. Rheumatology.

[33] Andrijevic I., Matijasevic J., Andrijevic L., Kovacevic T., Zaric B. (2014). Interleukin-6 and procalcitonin as biomarkers in mortality prediction of hospitalized patients with community acquired pneumonia. Ann. Thorac. Med..

[34] Naik A., Thakur N. (2024). Epigenetic regulation of TGF-beta and vice versa in cancers—A review on recent developments. Biochim. Biophys. Acta Rev. Cancer.

[35] Dandrea M., Donadelli M., Costanzo C., Scarpa A., Palmieri M. (2009). MeCP2/H3meK9 are involved in IL-6 gene silencing in pancreatic adenocarcinoma cell lines. Nucleic Acids Res..

[36] D’Cruz O.J., Qazi S., Hwang L., Ng K., Trieu V. (2018). Impact of targeting transforming growth factor beta-2 with antisense OT-101 on the cytokine and chemokine profile in patients with advanced pancreatic cancer. OncoTargets Ther..

[37] Jaschinski F., Rothhammer T., Jachimczak P., Seitz C., Schneider A., Schlingensiepen K.H. (2011). The antisense oligonucleotide trabedersen (AP 12009) for the targeted inhibition of TGF-beta2. Curr. Pharm. Biotechnol..

[38] Lee H.K., Nam M.W., Go R.E., Koo J., Kim T.H., Park J.E., Choi K.C. (2023). TGF-beta2 antisense oligonucleotide enhances T-cell mediated anti-tumor activities by IL-2 via attenuation of fibrotic reaction in a humanized mouse model of pancreatic ductal adenocarcinoma. Biomed. Pharmacother..

[39] Trieu V., Potts M., Myers S., Richardson S., Qazi S. (2025). TGFB2 Gene Methylation in Tumors with Low CD8+ T-Cell Infiltration Drives Positive Prognostic Overall Survival Responses in Pancreatic Ductal Adenocarcinoma. Int. J. Mol. Sci..

[40] Jiang X.M., Xu Y.L., Yuan L.W., Zhang L.L., Huang M.Y., Ye Z.H., Su M.X., Chen X.P., Zhu H., Ye R.D. (2021). TGFbeta2-mediated epithelial-mesenchymal transition and NF-kappaB pathway activation contribute to osimertinib resistance. Acta Pharmacol. Sin..

[41] Yang B., Bai J., Shi R., Shao X., Yang Y., Jin Y., Che X., Zhang Y., Qu X., Liu Y. (2020). TGFB2 serves as a link between epithelial-mesenchymal transition and tumor mutation burden in gastric cancer. Int. Immunopharmacol..

[42] Kim J., Lee T.S., Lee M.H., Cho I.R., Ryu J.K., Kim Y.T., Lee S.H., Paik W.H. (2024). Pancreatic Cancer Treatment Targeting the HGF/c-MET Pathway: The MEK Inhibitor Trametinib. Cancers.

[43] Zheng H., Yang X., Huang N., Yuan S., Li J., Liu X., Jiang Q., Wu S., Ju Y., Kleeff J. (2024). Chimeric antigen receptor macrophages targeting c-MET(CAR-M-c-MET) inhibit pancreatic cancer progression and improve cytotoxic chemotherapeutic efficacy. Mol. Cancer.

[44] Liu Z., Liang Q., Ren Y., Guo C., Ge X., Wang L., Cheng Q., Luo P., Zhang Y., Han X. (2023). Immunosenescence: Molecular mechanisms and diseases. Signal Transduct. Target. Ther..

[45] Li B., Dewey C.N. (2011). RSEM: Accurate transcript quantification from RNA-Seq data with or without a reference genome. BMC Bioinform..

[46] Bartha A., Gyorffy B. (2021). TNMplot.com: A Web Tool for the Comparison of Gene Expression in Normal, Tumor and Metastatic Tissues. Int. J. Mol. Sci..

