# Supplementary material for: TGFB2 Expression and Methylation Predict Overall Survival in Pancreatic Ductal Adenocarcinoma Patients
Source: Int J Mol Sci. 2025 Jul 1;26(13):6357. doi: 10.3390/ijms26136357 (PMC12250087; doi:10.3390/ijms26136357)
Supplement: Supplementary file 1 [file ijms-26-06357-s001.zip › ijms-3699666-supplementary.pdf]

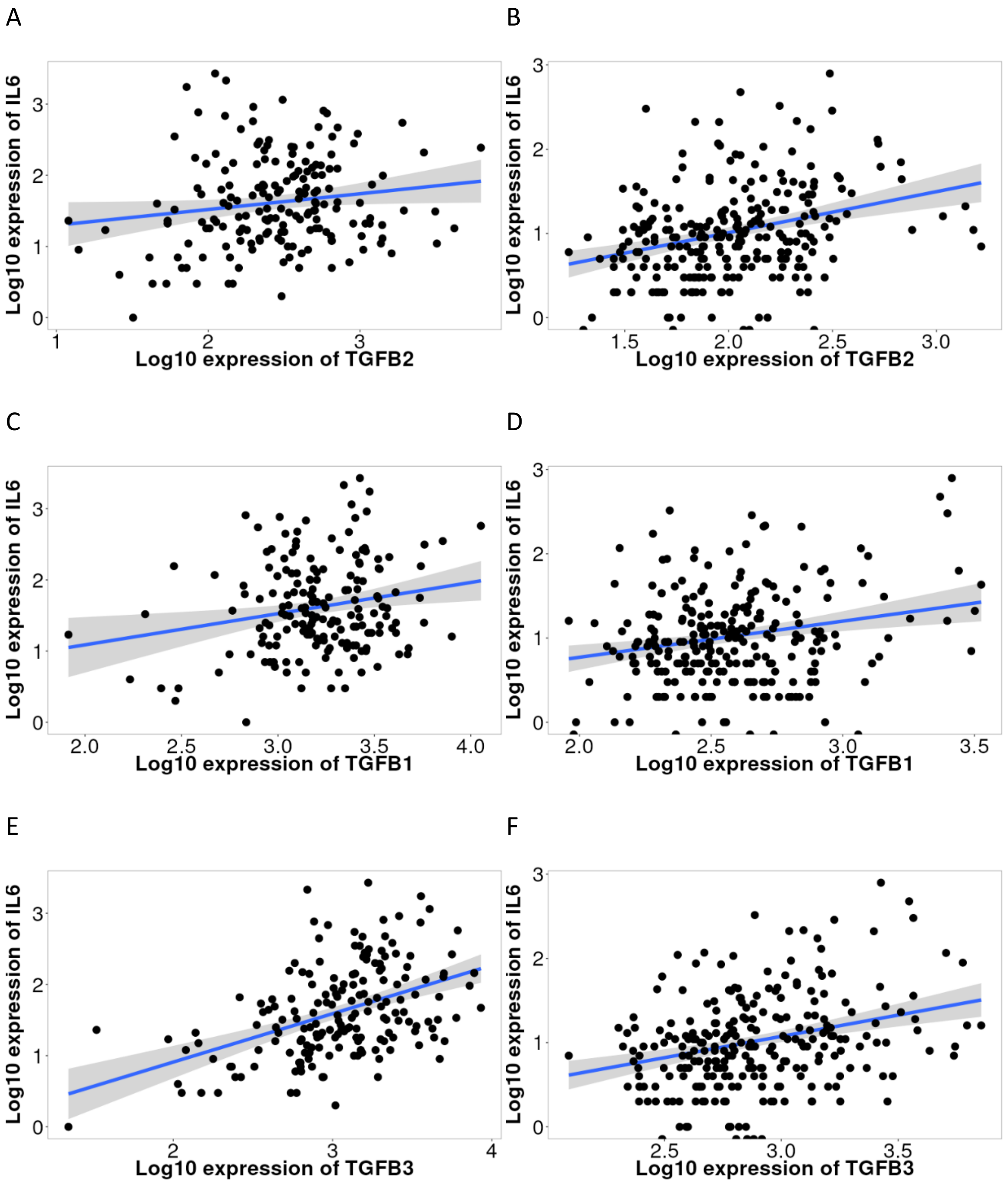

Figure S1. Correlation analysis between TGFB and IL6 expression in PDAC tumor and normal tissues.

(A) TGFB2 and IL6 mRNA expression in pancreatic tumor tissues did not show a significant correlation (R = 0.14). (B) TGFB2 and IL6 mRNA expression in normal tissues showed a significant correlation (R = 0.32). (C) TGFB1 and IL6 mRNA expression in pancreatic tumor

tissues did not show a significant correlation (R = 0.13). (D) TGFB1 and IL6 mRNA expression in normal tissues did not show a significant correlation (R = 0.13). (E) TGFB3 and IL6 mRNA expression in pancreatic tumor tissues showed a significant correlation (R = 0.44). (F) TGFB3 and IL6 mRNA expression in normal tissues did not show a significant correlation (R = 0.29).
